# The Challenge of Lorazepam Failure: Malignant Catatonia Treated Successfully with Valproate

**DOI:** 10.1192/j.eurpsy.2024.1526

**Published:** 2024-08-27

**Authors:** J. Kim, E. Garrels, A. Zamiri, K. Tran, A. Francis

**Affiliations:** ^1^Psychiatry; ^2^Bronxcare Health System, Bronx; ^3^Penn State College of Medicine, Hershey, United States

## Abstract

**Introduction:**

Despite the unclear nature of catatonia, the treatment response of catatonia to benzodiazepines is widely known for its typical, dramatic recovery. The neurobiological correlates of this phenomenon regarding specific receptors and neurotransmitters are unclear, as are the potential treatment options. This is important to consider when the most commonly recommended treatments of catatonia with Lorazepam or Electroconvulsive Therapy (ECT) are unavailable or unsuccessful. In this report, we describe a case of severe, malignant catatonia and psychosis mostly unresponsive to Lorazepam during two different hospitalizations, but with eventual return to baseline after successful treatment with Valproate.

**Objectives:**

- To describe a unique case of malignant catatonia that was unresponsive to Lorazepam
- To illustrate the potential utility of Valproate as an alternative treatment strategy for catatonia

**Methods:**

This is a case report.

**Results:**

A 19-year-old Hispanic male presented to our hospital initially with family reports of severe and sudden depression with bizarre behavior. Prior to this admission, the patient had been discharged recently from another tertiary hospital following a 2-week admission for severe catatonia. Chart review from that admission scored the patient’s Bush-Francis Catatonia Rating Scale (BFCRS) at 16, which remained mostly unchanged after numerous additional intramuscular doses and standing oral doses of Lorazepam, with a reduction of BFCRS the next day of only 2. During the patient’s admission at our hospital, the patient endorsed bizarre, guilt-related delusions, and his catatonia was more severe and malignant with a BFCRS of 19, with tachycardia and diaphoresis. The patient was initially given a total of seven doses of a mix of intramuscular and oral Lorazepam (total 18mg), with a minimal 2-point reduction in BFCRS. As ECT was unavailable, Lorazepam was discontinued in favor of a trial of oral Valproate 500mg twice daily, and after his catatonia subsided (with a serum level of 60.8), he was started on oral Risperidone 0.5mg once at night, titrated up to 3mg twice daily, and eventually returned to baseline as confirmed by his family members.

**Conclusions:**

The treatment of catatonia with Lorazepam is usually reliable and has been found to be up to 80% effective, but when the recommended use of benzodiazepines and ECT fail or are unavailable, there are few studies exploring the viability of alternative treatment options. With the use of Valproate, previous studies have shown it can treat even severe catatonia (KrÜger, J Neuropsychiatry 2001; 13:303-304), or can actually be its cause (Lauterbach, Neuropsychiatry, Neuropsychology, and Behavioral Neurology. 1998 Jul;11(3):157-163). As such, this case report highlights the importance of exploring alternative treatments for catatonia, including Valproate, in order to better tailor the management of this unique syndrome.

**Disclosure of Interest:**

None Declared

